# *AbhemC* encoding porphobilinogen deaminase plays an important role in chlorophyll biosynthesis and function in albino *Ananas comosus* var. *bracteatus* leaves

**DOI:** 10.7717/peerj.11118

**Published:** 2021-03-30

**Authors:** Yanbin Xue, Xia Li, Meiqin Mao, Yehua He, Mark Owusu Adjei, Xuzixin Zhou, Hao Hu, Jiawen Liu, Xi Li, Jun Ma

**Affiliations:** 1 College of Landscape Architecture, Sichuan Agricultural University, Chengdu, China; 2 College of Biology and Food Engineering, Chongqing Three Gorges College, Chongqing, China; 3 South China Agricultural University, Guangzhou, China

**Keywords:** *AbhemC*, Chlorophyll biosynthesis, Albino, Genetic transformation, Gene function identification

## Abstract

**Background:**

The chimeric leaves of* Ananas comosus* var. *bracteatus* are composed of normal green parts (Grs) and albino white parts (Whs). Although the underlying mechanism of albinism in *A. comosus* var. *bracteatus* leaves is not fully understood, it is likely associated with the chlorophyll (Chl) biosynthesis. In this biosynthetic process, porphobilinogen deaminase (PBGD) plays a crucial role by catalyzing the conversion of porphobilinogen (PBG) to uroporphyrinogen III (Urogen III). Therefore, its encoding gene *AbhemC* was investigated here in association with Chl biosynthesis and albinism in chimeric *A. comosus* var. *bracteatus* leaves.

**Methods:**

The Chl content, main Chl biosynthesis precursor content, and main enzyme activity were determined and compared between the Whs and Grs of *A. comosus* var. *bracteatus* leaves. In addition, *AbhemC* was cloned and its transcriptional expression and prokaryotic protein expression were analyzed. Furthermore, RNAi-mediated silencing of *AbhemC* was produced and assessed in tobacco plants.

**Results:**

The concentration of Chl a and Chl b in the Grs was significantly higher than that in the Whs, respectively. Additionally, the content of the Chl biosynthesis precursor Urogen III decreased significantly in the Whs compared with the Grs. Thus, the transition of PBG to Urogen III may be the first rate-limiting step leading to albinism in the chimeric leaves of *A. comosus* var. *bracteatus*. The gene *AbhemC* comprised 1,135 bp and was encoded into a protein with 371 amino acids; phylogenetically, *AbhemC* was most closely related to *hemC* of pineapple*.* Prokaryotic expression and *in vitro* enzyme activity analysis showed that the cloned mRNA sequence of *AbhemC* was successfully integrated and had PBGD activity. Compared with control plants, transgenic tobacco leaves with pFGC5941-*AbhemC*-RNAi vector were substantially less green with significantly reduced *hemC* expression and Chl content, as well as reduced PBGD enzyme activity and significantly decreased content of Chl biosynthesis precursors from Urogen III onwards. Our results suggest that the absence of *hemC* expression reduces the enzyme activity of PBGD, which blocks the transition of PBG to Urogen III, and in turn suppresses Chl synthesis leading to the pale-green leaf color. Therefore, we suggest that *AbhemC* plays an important role in Chl synthesis and may be an important factor in the albinism of *A. comosus* var. *bracteatus* leaves.

## Introduction

Bromeliads are perennial evergreen herbs of the family *Bromeliaceae* which are native to South America; and var. *bracteatus* belongs to one of five varieties of *Ananas comosus* (Coppensd’Eeckenbrugge & Leal, 2003). Chimeric *A. comosus* var. *bracteatus* is cultivated commercially as an important new ornamental plant for its colorful leaves and strange fruit. The high quality silk fiber and a large number of secondary metabolites that produce in the stem and leaves of it are widely used (Collins, 1960; Montinola, 1991). The chimeric leaves consist of normal green cells and albino white cells. In contrast to other albino mutants, these chimeric plants can survive normally; therefore, they provide a good model for the study of albinism. We previously performed comparative transcriptomic analyses of complete green leaves and complete white leaves of *A. comosus* var. *bracteatus* shoots, which were derived from chimeric var. *bracteatus* by tissue culture, we showed that differences at the transcriptional level were associated with photosynthetic pigment synthesis and chloroplast development, which in turn might be responsible for differences in leaf color (Li et al., 2017). However, beyond this initial research, little is known about the molecular mechanism of albinism in *A. comosus* var. *bracteatus*.

In higher plants, leaf color formation is influenced by photosynthetic pigments and anthocyanin (Liu, Chang & Du, 2015). The biosynthesis of Chl in higher plants are performed and accomplished by sequential reactions. In the common steps, the synthesis of heme and Chl starts with *δ*-aminolevulinic acid (ALA) as the first precursor for the synthesis of all tetrapyrroles. Briefly, the bimolecular ALA is condensed, and this sequential step reaction requires the catalysis of ALA dehydratase (ALAD) to synthesize the porphobilinogen (PBG) (Mills-Davies et al., 2016). Subsequently, the hydroxy-methylbilane (Hmb) is formed by four molecular PBG catalyzed by PBG deaminase encoded by *hemC* gene, which is the object of this study. PBGDs have been isolated from both prokaryotic and eukaryotic organisms, including *E. coli* (Jordan & Warren, 1987), plants (Roberts et al., 2013) and mammals (Gill et al., 2009). *HemC* gene encoding PBGD enzyme has been cloned for the first time in *E. coli* (Thomas & Jordan, 1986).The acetyl and propionyl groups of the d-porphyrin ring were isomerized to form uroporphyrinogen III (Urogen III) (Pryde & Scott, 1979; Jordan & Berry, 1980). After decarboxylation of the side chains of the porphyrin ring, coproporphyrinogen III (Coprogen III) is formed and then protoporphyrin IX (Proto IX) is formed after oxidation. Mg^2+^ is chelated onto protoporphyrin IX to form Mg-protoporphyrin IX (Papenbrock et al., 2000),which forms Mg-protoporphyrin IX monomethyl ester  by methyltransferase methylation (Alawady & Grimm, 2005). The late steps of Chl synthesis include the transformation of light-dependent Pchlide a to chlorophyllide, the formation of Chl a, and finally the production of Chl b (Fujita, 1996; Reinbothe & Reinbothe, 1996; Heyes & Hunter, 2005; Pattanayak et al., 2005; Rudiger et al., 2005). Chl is distributed in chloroplast thylakoid membrane. Chl biosynthesis occurs in parallel with chloroplast development, which is essential for photosynthesis. The development of chloroplasts, the number and size of chloroplasts directly affect the color and photosynthetic efficiency of leaves.

In our previously published work, we hypothesized that PBGD catalyses PBG to UROS transformation as a rate-limiting step in chlorophyll synthesis through transcriptomic data and Chl precursor content in CWh/CGr, and the *AbhemC* gene encoding PBGD may be a key gene for chlorophyll synthesis (Li et al., 2017). In this study, we further demonstrated the function of AbHEMC through RNAi transformation of tobacco. We successfully found the conversion of PBG to Urogen III in Chl biosynthesis were the rate-limiting steps in leaves of chimeric *A. comosus* var. *bracteatus*. We discovered this by comparing the main precursors of Chl in the albino white parts of leaves with those in the normal green parts. Specifically, the gene *AbhemC*, which encodes PBGD, was cloned and its sequence and gene transcription were analyzed. Prokaryotic expression of the AbHEMC protein was derived and in vitro PBGD activity was verified. A pFGC5941-*AbhemC*-RNAi expression vector was constructed and used to transform to tobacco to verify the suppression of the *hemC* expression, which reduced PBGD protein activity and further inhibited the transition of PBG to Urogen III. The resultant lack of Urogen III reduced the content of specific precursors in Chl biosynthesis, which in turn significantly reduced Chl content and resulted in pale green-colored leaves. Therefore, this research indicates the role of *AbhemC* in Chl biosynthesis and albinism in *A. comosus* var. *bracteatus.*

## Materials & Methods

### Plant materials

**Figure 1:**
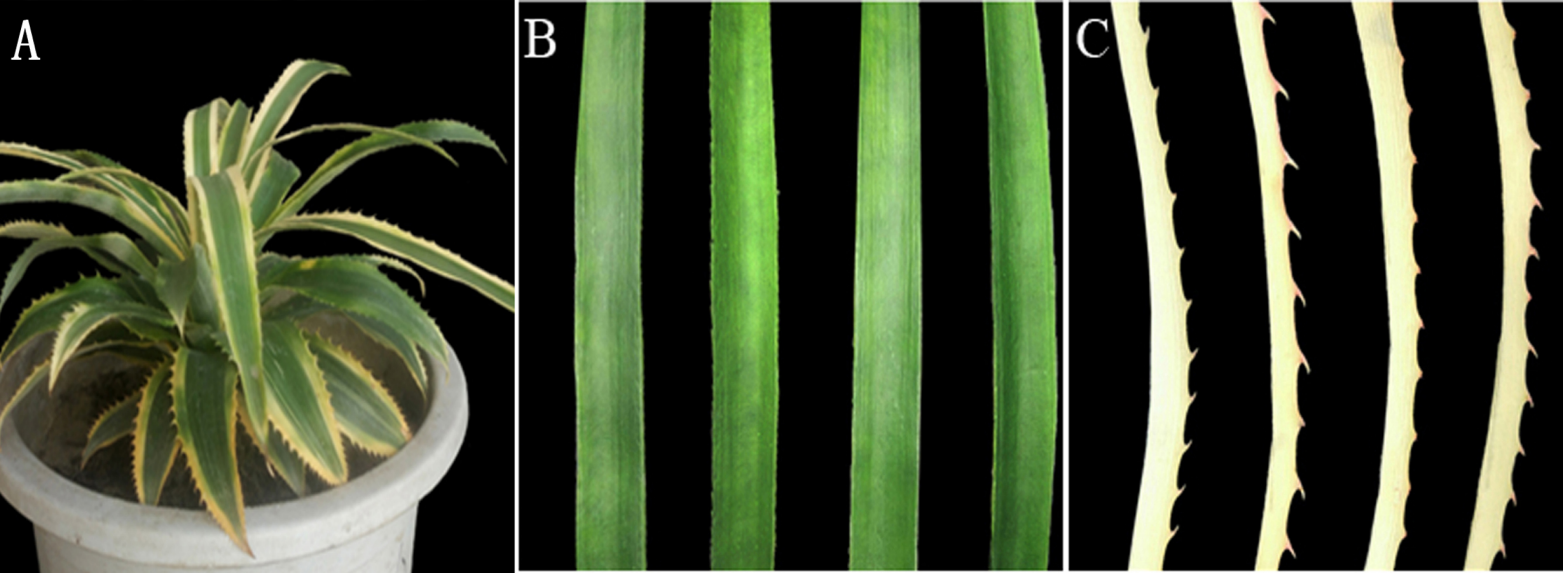
Plant materials used in this study. (A) Chimeric plant of *A. comosus* var. *bracteatus*. **(**B) The green parts of the chimeric leaves. (C) The white parts of the chimeric leaves.

Two-year-old chimeric *A. comosus* var. *bracteatus* plants were generated from the crown buds of the mother plant and grown at the experimental site of Sichuan Agricultural University.The mother plants were purchased from a supplier in Zhanjiang city, Guangdong province, China (21°12′N, 110°24′E). They were then grown at a temperature of 20 °C−30 °C during the day and 15 °C−18 °C at night, with 60–80% relative humidity. The normal green parts (Grs) and albino white parts (Whs) of the leaves were used in this study ( Fig. 1).

### Measurement of photosynthetic pigments and synthetic precursors, and ALAD, PBGD, and uroporphyrinogen III synthase enzyme activity

The Whs and Grs of the chimeric leaves were used to determine Chl content and measure Chl biosynthetic precursors. Previously described methods were used to determine the contents of Chl a and Chl b (Holm, 1954), ALA (Dei, 1985), PBG (Bogorad, 1962), and Urogen III and Coprogen III (Czarnecki, Peter & Grimm, 2011). In addition, the contents of Proto IX, Mg-protoporphyrin IX (Mg-Proto IX), and protochlorophyllide (Pchlide) were assessed according to protocols reported by Rebeiz et al. (1975) and Lee et al. (1992). To measure enzyme activity, the following methods were applied: that of Mauzerall & Granick (1958) for ALAD, that of Lee et al. (1992) for PBGD, and that of Li et al. (2017) for uroporphyrinogen III synthase (UROS). Three independent biological replicates were used when measuring Chl a, Chl b, and the main Chl biosynthetic precursors, and the major enzyme activity.

### Cloning and bioinformatics analysis of *AbhemC*

Specific primer pairs of *AbhemC*-F and *AbhemC*-R (Table S1) were designed according to the transcriptome sequence results (Ma et al., 2015) to amplify the full-length sequence of *hemC* from the leaves of *A.comosus* var. *bracteatus*. The ORF Finder (open reading frame finder) program was used to predict the ORF (http://www.ncbi.nlm.nih.gov/gorf/gorf.html), for which the conserved domains were obtained using NCBI resources (http://www.ncbi.nlm.nih.gov/Structure/cdd/wrpsb.cgi and http://smart.embl-heidelberg.de/index2.cgi), and the secondary structure was predicted using PSIPRED (http://bioinf.cs.ucl.ac.uk/psipred/). A three-dimension image of the protein and the multiple sequence alignment prediction were performed using SWISS-MODEL (http://swissmodel.expasy.org) and CLUSTX , respectively. The functional structure of the protein was predicted using InterProScan (http://www.ebi.ac.uk/interpro/search/sequence/) and ScanProsite (http://us.expasy.org/prosite) (Zdobnov & Apweiler, 2001). MEGA 5.0 was used with the neighbor-joining method to construct a phylogentic tree (Tamura et al., 2011).

### RNA extraction and real-time quantitative PCR analysis

Total RNA from the Grs and Whs of chimeric leaves was carried out separately using TRIzol reagents (Invitrogen, USA ) following the recommendations of the manufacturer. The RNA was then reverse transcribed to cDNA using a PrimeScript RT Reagent kit. Using PCR system ABI prism 7900 Real-Time, the relative expression of *AbhemC* and *hemC* of tobacco plant were measured with SYBR Premix Ex TaqTM Kits (Takara), where *18S rRNA* and *Actin* Tobacco housekeeping gene were used as an endogenous controls respectively (Li et al., 2014). The PCR amplification system was carried out as follows, heating for 30s at 95 °C, 40 cycles of denaturation at 95 °C for 15 s, annealing for 31 s at 58 °C, and extension at 72 °C for 35 s. Triplicate quantitative PCR experiments were performed for each sample, and the expression values obtained were normalized against the 18S rRNA gene. Data analysis of the relatively expressed gene was conducted using the Pfaffl method (Pfaffl, 2001). All the reactions were performed with three biological replicates. Primer pairs of *AbhemC-1*, *Actin* and *hemC-1* were detailed in Table S1.

### Prokaryotic expression of AbHEMC protein

After digestion with *NdeI* and *XhoI*, the full-length sequences of *AbhemC* were sub-cloned into pET15b vector to express the fusion protein. The purification of the protein involved ultrasonic fragmentation and nickel agarose affinity chromatography. The ultrasonic crushing of bacteria was performed in an ice bath twice at 20 min per session, with ultrasonic 2S suspended for 6 s as a cycle. Nickel agarose affinity chromatography was performed after ultrasound chromatography to purify the protein. The recombinant protein was expressed in *E. coli* Rosetta-gami (DE3) following the protocol of Ma et al. (2012), and its enzymatic activity was detected using the method of Lee et al. (1992).

### Vector construction and tobacco transformation

**Figure 2:**
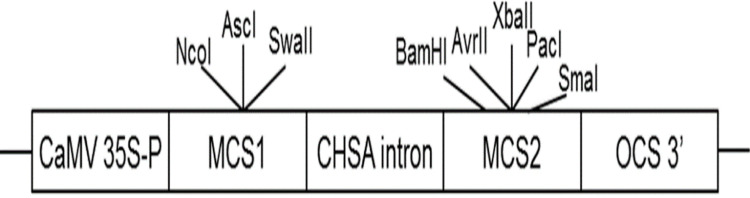
Construction of interference vector pFGC5941-*AbhemC*-RNAi. MCS1, Multiple cloning site1; MCS2, Multiple cloning site2.

*AbhemC* is highly homologous with *hemC* of tobacco; therefore, for the RNAi construct, the common conservative region of the two genes was amplified using primers for *hemC-2* ( Table S1 ) and then subcloned into the binary vector pFGC5941. The pFGC5941 plasmid was then used to construct the RNAi vector (Deng et al., 2013). Conserved fragments were ligated into multiple cloning site 1 (MCS1) using *NcoI* and *SwaI* in the forward direction and into MCS2 using *SmaI* and *XbaI* in the reverse direction (Fig. 2). The recombinant plasmid was cloned into Agrobacterium strain EHA105 and then it was used to transform tobacco by Agrobacterium mediated method. The transformation of pFGC5941-AbhemC-RNAi vector to tobacco was conducted according to the protocol of Horch et al. (1985). Subsequently, the transgenic plants were screened and identified by the herbicide (PPT)-resistance gene *Bar* in the pFGC5941 vector. PCR identification of transgenic tobacco was performed using genomic DNA of resistant tobacco as template, wild-type tobacco genomic DNA template as negative control, and pFGC5941 plasmid as positive control. Physiological indicators were determined by analysis of three technical replicates, each of which was taken from three different transgenic plants.

### Statistical analysis

SPSS was used as a statistical platform for the analysis of Chl contents ,precursors of Chl and enzyme activity. Two independent samples T test were used for statistical analysis.Figures were made with Excel 2016 in our experiments.

## Results

### Assessment of Chl biosynthesis in the Whs and Grs of the chimeric leaves

**Figure 3:**
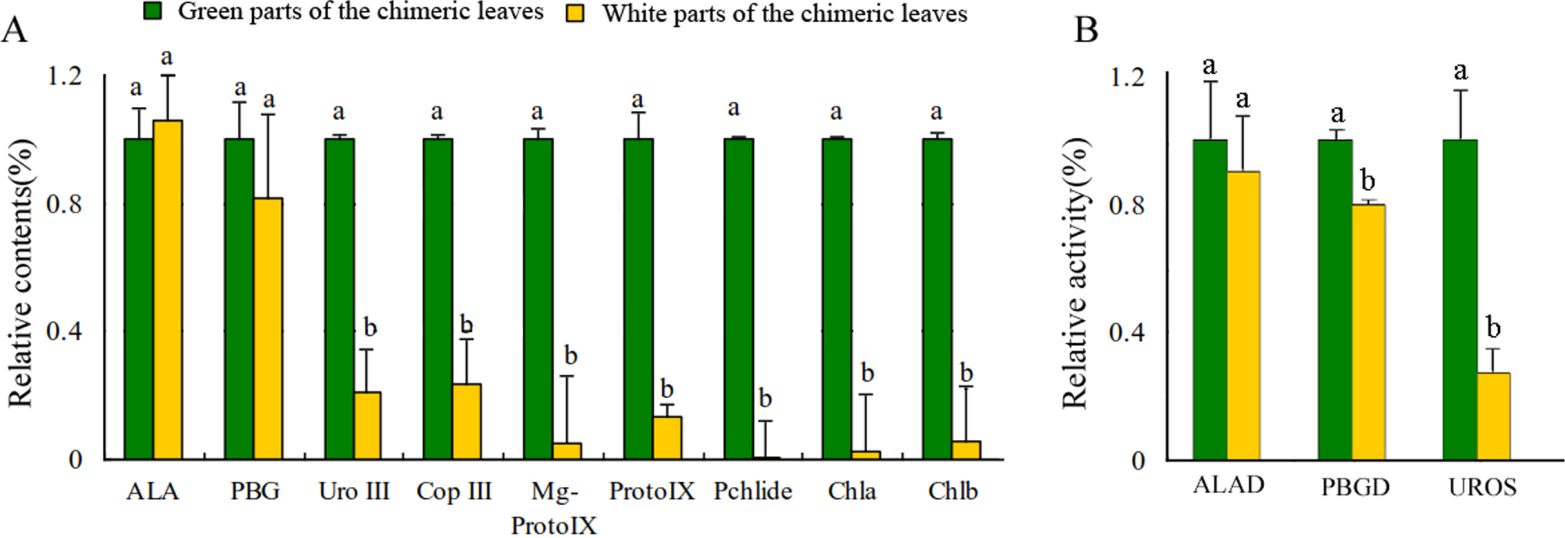
Analysis of the Chl biosynthesis in the white and green parts of the chimeric leaves. (A) The variation of relative amount of main Chl precursors; (B) Enzymes activity of ALAD, PBGD and UROS between Whs and Grs of the chimeric leaves. Different lowercase letters on the same line indicate significant differences at a level of *P* < 0.05.

Leaf color is usually associated with Chl contents (Kim & An, 2013). The concentrations of Chl a and Chl b in the Grs were significantly higher than those in the Whs of the chimeric leaves, respectively (Fig. 3A). To find out the key step of Chl biosynthesis in Whs which caused the albino of the leaves, the contents of the main Chl biosynthesis precursors were evaluated. Although there was no significant difference in the ALA and PBG content of the Whs and Grs of chimeric leaves, the content of Urogen III, Coprogen III, Proto IX, Mg-proto IX, and Pchlide was significantly lower in the Whs (Fig. 3A). These changes of the precursors suggested that the conversion of PBG to Urogen III was the first speed-limiting step in Chl biosynthesis in the Whs. The transition of ALA to PBG is catalyzed by ALAD, while that of PBG to Urogen III is catalyzed by PBGD and UROS. The enzymes activity of ALAD, PBGD and UROS are shown in Fig. 3B. The ALAD activity did not differ between the Whs and Grs, but the enzymes activity of PBGD and UROS were significantly reduced in the Whs. This suggests that decreased PBGD and UROS activity may have suppressed Urogen III formation in the Whs of leaves. The *hemC* gene encoding PBGD, which catalyses polymerization of PBG to produce 1-hydroxymethylbilane, may be the key functional gene in Chl biosynthesis and may play a role in the albino phenotype of chimeric *A. comosus* var. *bracteatus*.

### Cloning and bioinformatics analysis of *AbhemC*

**Figure 4:**
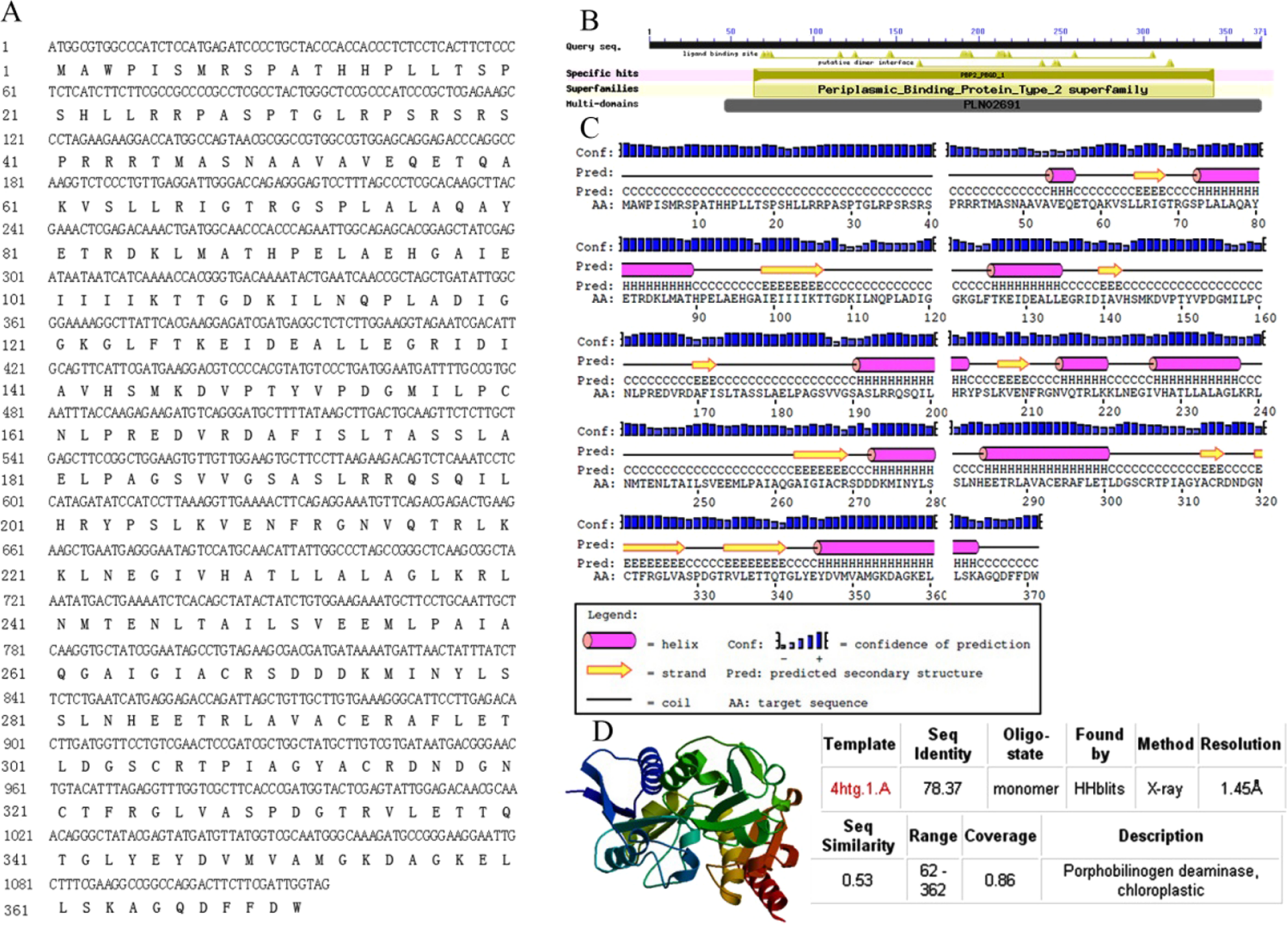
Sequence analysis of *AbhemC.*. (A) The *AbhemC* sequence and its encoded protein; (B) the conserved domains searched by NCBI; (C) the secondary structure of AbHEMC predicted protein. (D) The 3D structure of AbHEMC protein.

**Figure 5:**
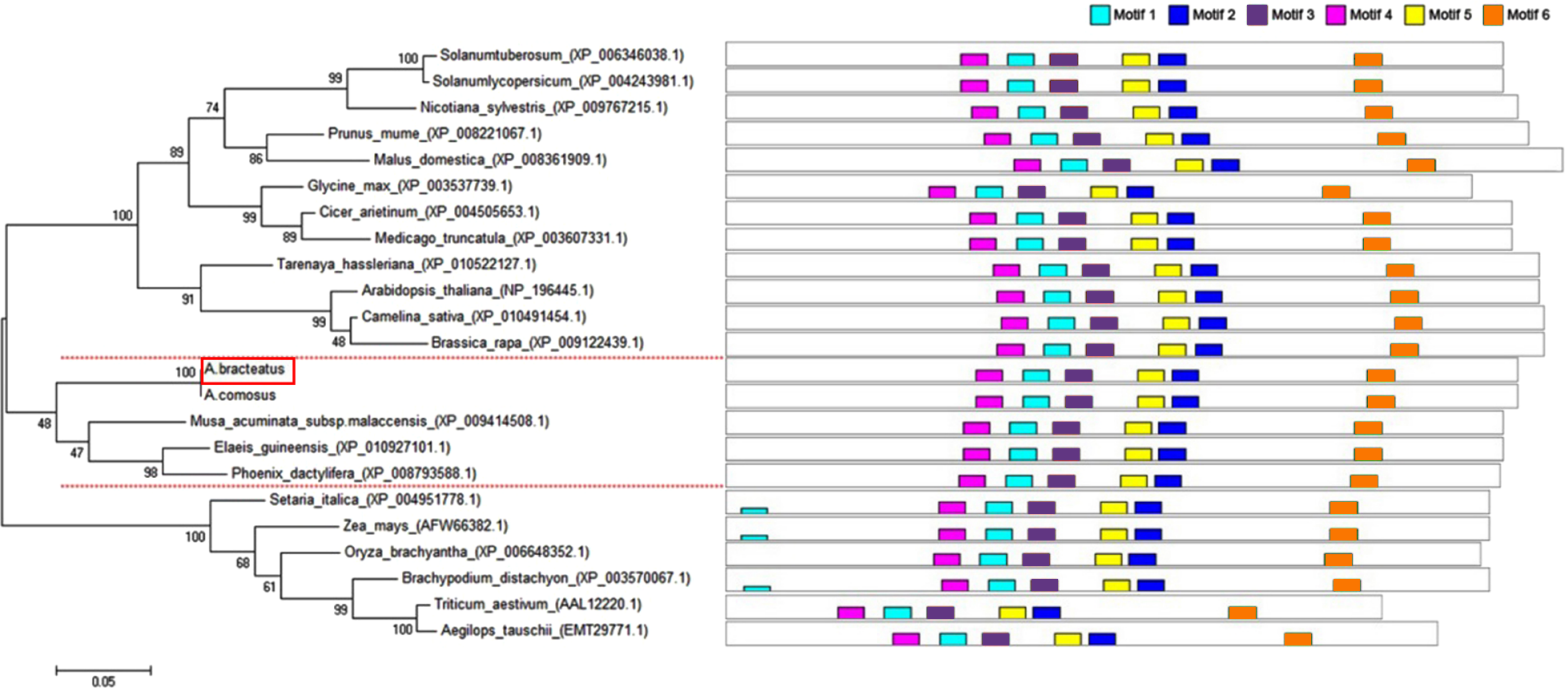
Phylogenetic tree of HEMC family proteins based on the full-length amino acid sequences. The red dashes lines marked the three clades of the phylogenetic tree. Multiple sequences alignment of predicted amino acid sequences of HEMC family protein.

The full-length sequence of *AbhemC* was amplified, and the amplification products were purified, cloned and sequenced. The mRNA sequence obtained was determined to be that of *AbhemC* (GenBank accession number: KT377022); it comprised1135bp with an ORF of 1116bp. In addition, the AbHEMC protein was found to contain 371 amino acids; it belongs to the PBGD family (PLN02691) and had a high identity (78–82%), as well as a theoretical pI of 7.12 and molecular mass of 40.4 kD. The conserved domains of AbHEMC were identified using the NCBI Conserved Domain Search Service (Fig. 4B), and an uncharacterized subgroup of the PBGD family, namely the type 2 periplasmic binding protein fold (PBP2_PBGD_1), was found. Nine helices and nine strand structures were identified in the predicted secondary structure (Fig. 4C). Its 3D structure predicted a high sequence identity (78.37%) and similarity (0.53), and sequence coverage reached 0.86 with PBGD protein of *Arabidopsis thaliana* (Fig. 4D). Phylogenetic tree constructed with Molecular Evolutionary Genetics Analysis (MEGA) 5.0 showed that the phylogenetic tree was divided into three clades: temperate monocotyledons, tropical monocotyledons, and dicotyledons. It showed that AbHEMC was most closely related to AcHEMC in pineapple (Fig. 5). The predicted AbHEMC proteins of 21 other plants were derived from GenBank for phylogenetic analysis.

### Expression of *AbhemC* in the Whs and Grs of the chimeric leaves

**Figure 6:**
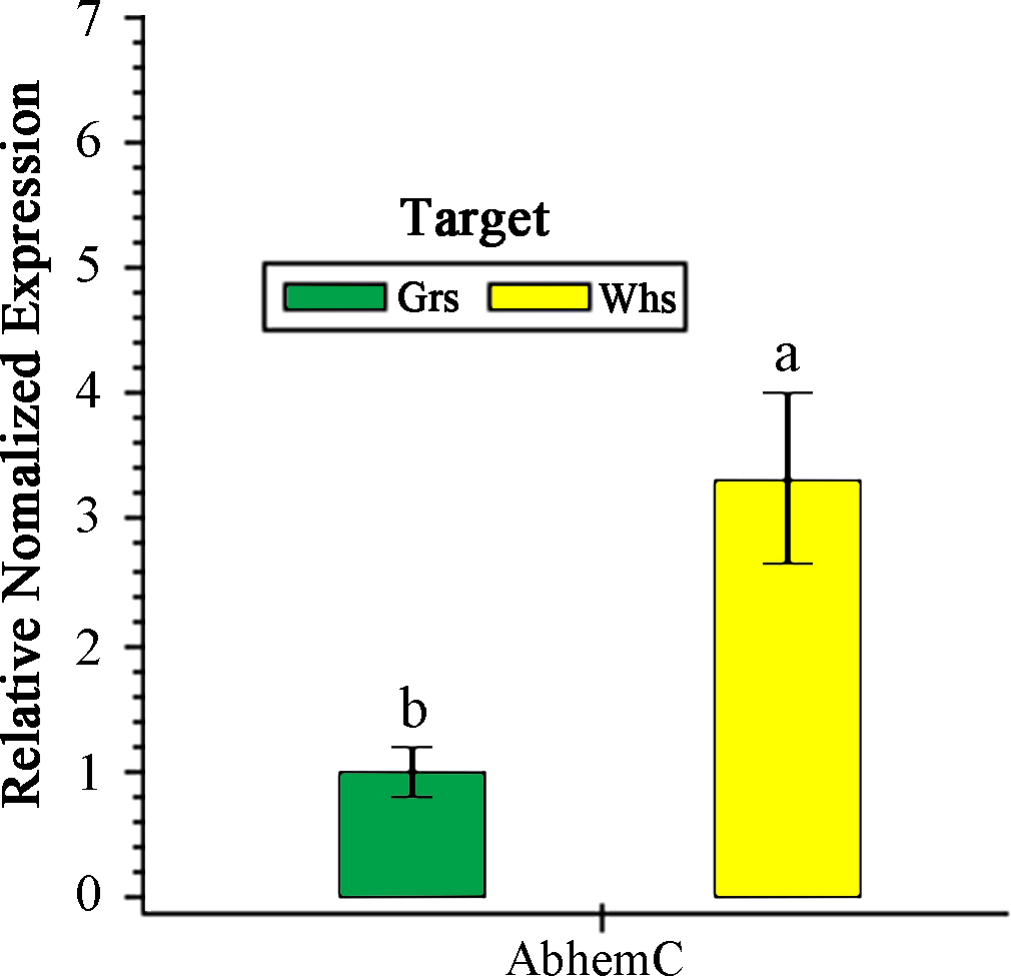
RT- qPCR analysis of *AbhemC* expression in in the white and green parts of the chimeric leaves. The relative value of *AbhemC* in Whs use the value of Grs as control and calculated as 1. Different letters in columus indicate statistically significant differences (*P* < 0.01).

Expression of *AbhemC* in the Whs was 3.32-fold greater than in the Grs (Fig. 6). In previous transcriptomic (RNA-Seq) analysis, Xue et al. (2019) showed that *hemC* was significantly upregulated in the Whs of chimeric leaves from *A. comosus* var. *bracteatus* when compared with expression in the Grs, consistent with the results of qPCR. In the present study, *AbhemC* expression was upregulated whereas PDGD enzyme activity was reduced in the Whs. This indicates that the reduced function of the *AbhemC*-encoded PBGD protein disrupted Chl synthesis. Furthermore, it suggests regulation of the PBGD enzyme activity at the protein level or at the level of translation.

### Prokaryotic expression and enzyme activity analysis of the AbHEMC protein

**Figure 7:**
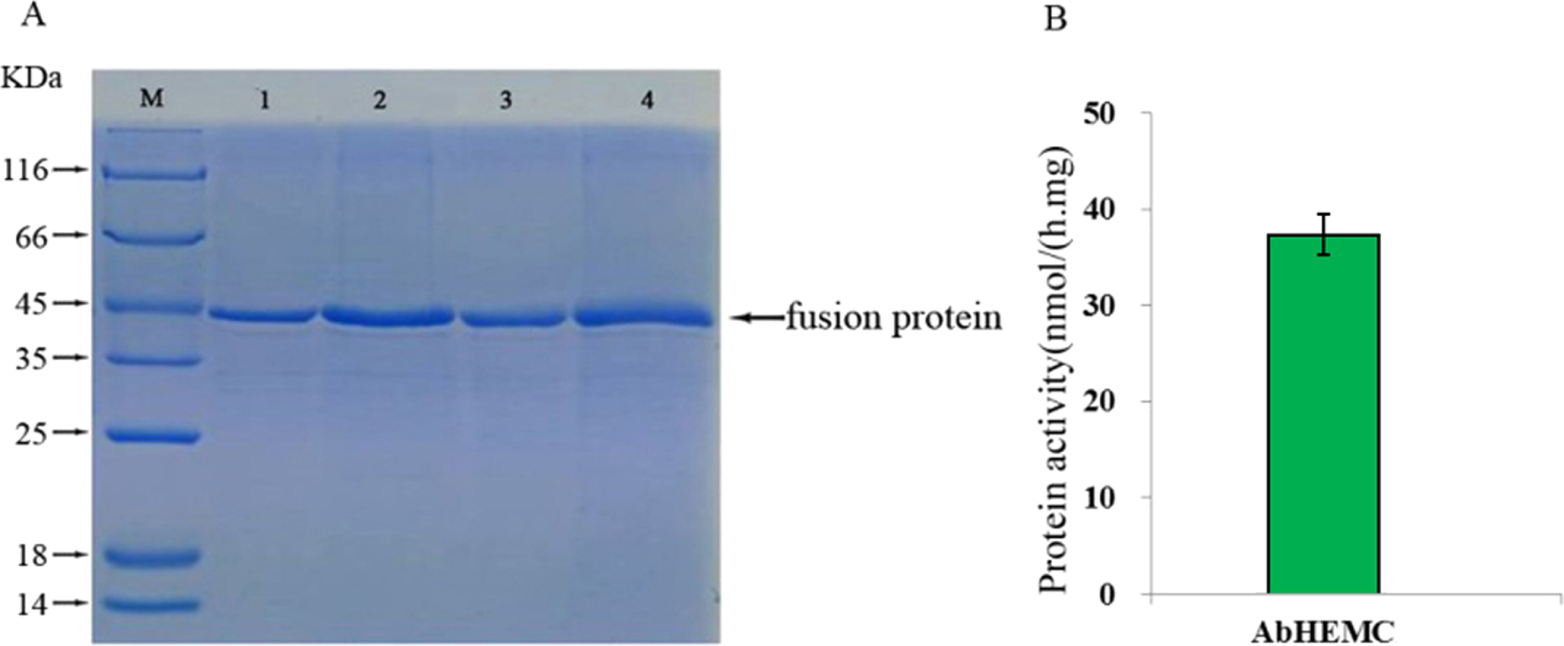
Prokaryotic expression and enzyme activity analysis of AbHEMC protein. (A) Analysis of pET-hemC protein expression by SDS-PAGE. Lane 1–4, Target proteins; M, protein molecular weight marker. (B) In vitro PBGD activity of prokaryotic expressed AbHEMC protein.

To verify the integrity of the mRNA sequence of the cloned *hemC* gene and the activity of the HEMC protein it encoded, prokaryotic expression and enzyme activity analysis were conducted. The ORF of *AbhemC* sequence was cloned into the expression vector pET-15b and expressed in *E. coli* Rosetta-gami (DE3) after induction with IPTG. SDS-PAGE analysis showed that the AbHEMC protein was expressed in *E. coli* and an obvious single band around 45 kD (lane 1–4 induced) was consistent with the expected molecular mass of AbHEMC (Fig. 7A). This confirmed that the mRNA sequence of *AbhemC* had been integrated and that it could translate a soluble fusion protein in *E. coli* cells. Further in vitro analysis showed that the prokaryotic-expressed AbHEMC protein had PBGD activity (Fig. 7B).

### Functional analysis of AbhemC by transformation

**Figure 8:**
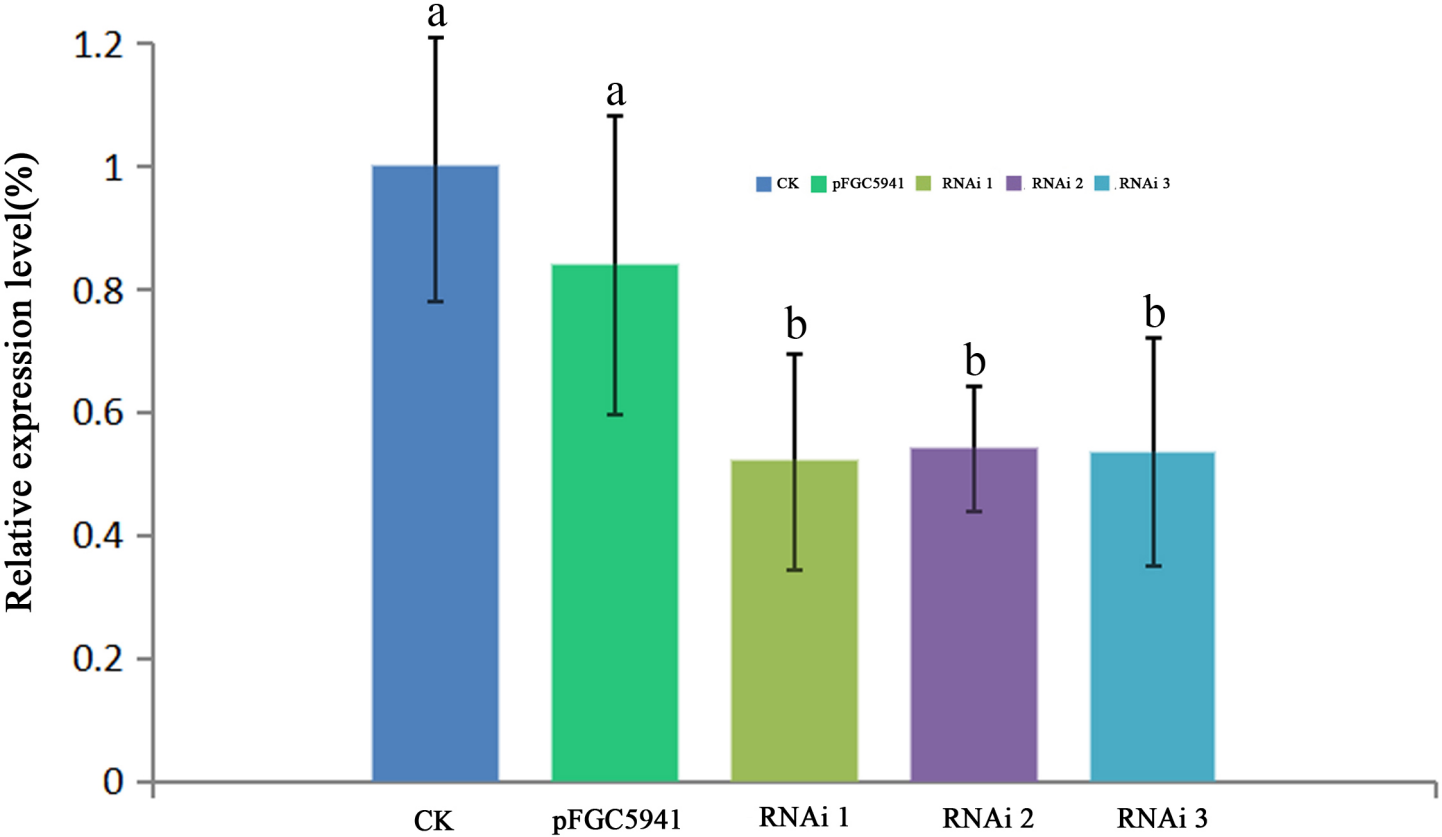
RT- qPCR analysis of *hemC* expression in three transgenic tobacco lines. The relative values of *AbhemC*-RNAi and pFGC5941 leaves which use the value of Wild type leaves as control and calculated as 1. Different letters in columns indicate statistically significant differences (*P* < 0.01) according to a *T*-test.

To identify the function of AbHEMC, the RNA interference (RNAi) expression analysis was carried out. *Agrobacterium* EHA105 and a pFGC5941-*AbhemC*-RNAi fusion plasmid were transformed in tobacco leaves. The pFGC5941 vector in tobacco plants was used as a positive control while wild tobacco plants were used as a negative control. The resistant tobacco DNA obtained from PPT screening was extracted and identified by PCR with specific primers of *Bar* gene on pFGC5941 vector. The size of the target band amplified by PCR product electrophoresis was about 500bp, which was consistent with the expectation. Therefore, these resistant tobacco were identified as positive transgenic tobacco. According to RT-qPCR analysis of the tobacco plants, transformation of the pFGC5941 vector did not influence the expression of *hemC* significantly, whereas transformation of pFGC5941-*AbhemC*-RNAi significantly suppressed *hemC* expression (Fig. 8).

**Figure 9:**
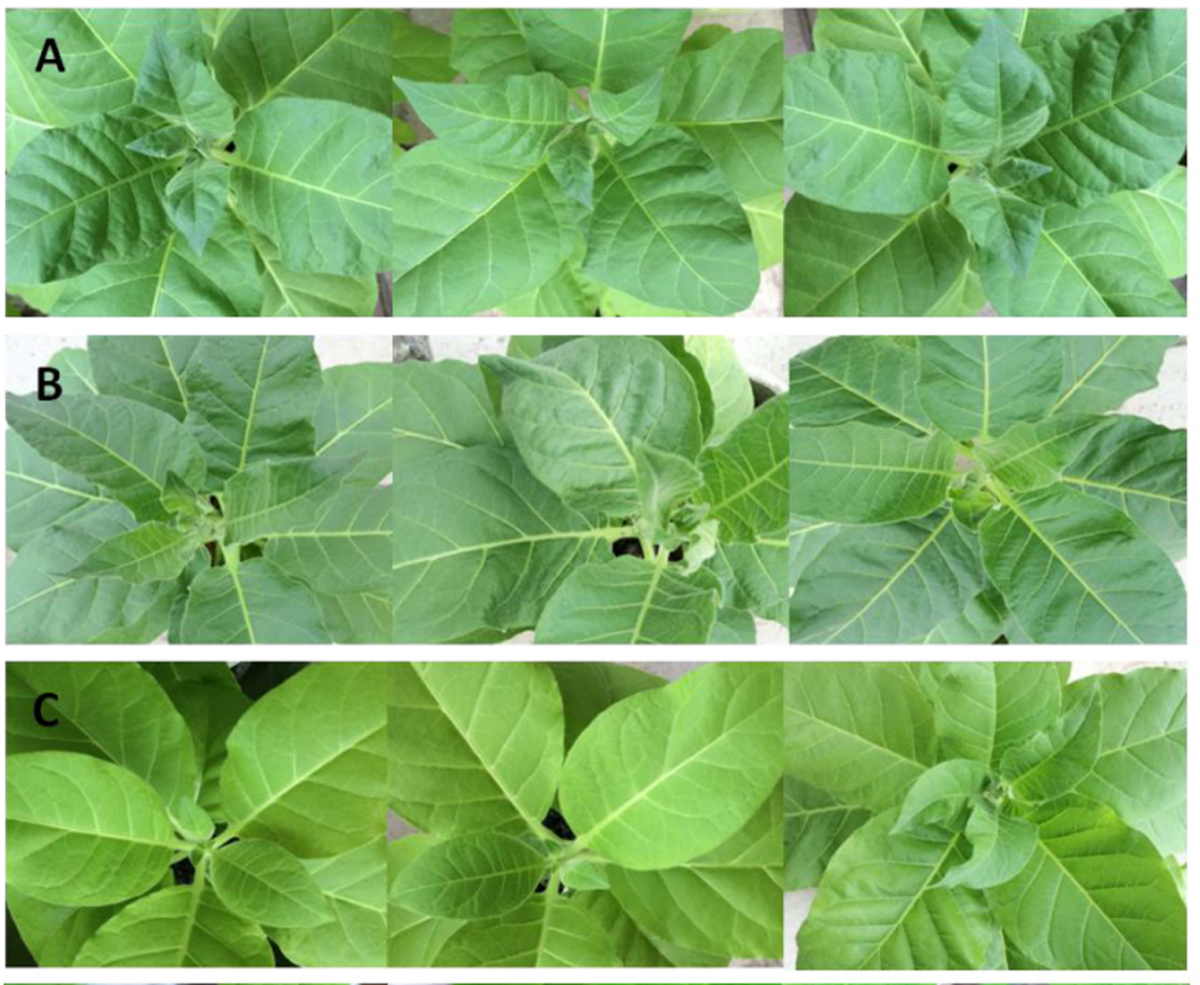
Leaf color of transformed and wild-type tobacco. (A) Wild type tobacco; (B) tobacco transformed with pFGC5941 vector; (C) tobacco transformed with pFGC5941-*AbhemC*-RNAi vector.

**Figure 10:**
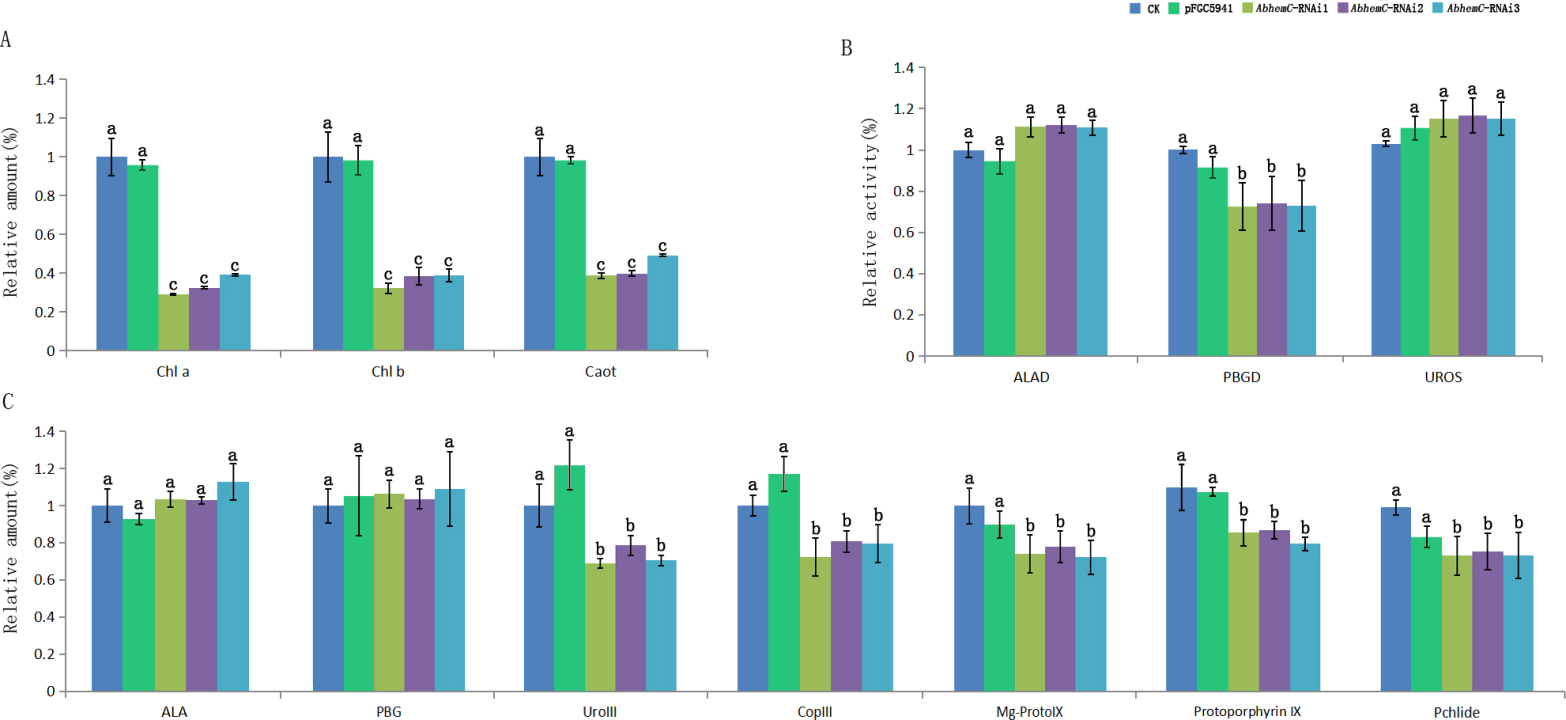
Determination of Chl content and related metabolites in tobacco. (A) The variation of relative amount of Chl and carotenoid in three types of tobacco; (B) the protein activity of ALAD, PBGD and UROS in three types of tobacco; (C) relative concentration of the precursors of Chl biosynthesis in three types of tobacco. Different letters in columns indicate statistically significant differences (*P* < 0.01) according to a *T*-test.

Furthermore, the leaf color of pFGC5941 vector-transformed tobacco plants closely resembled that of wild type tobacco plants, while the leaves of these two types were greener than the leaves of tobacco transformed with pFGC5941-*AbhemC*-RNAi vector (Fig. 9). In addition, the concentrations of Chl a and Chl b in tobacco plants transformed with pFGC5941-*AbhemC*-RNAi were significantly reduced relative to those in the wild type and pFGC5941 vector-transformed plants (Fig. 10A). These results indicate that Chl biosynthesis was significantly suppressed in tobacco plants transformed with the pFGC5941-*AbhemC*-RNAi vector. Thus, *hemC* is apparently a key gene in the Chl metabolism pathway and suppression of its expression inhibits Chl synthesis.

The enzyme activity of ALAD, PBGD, and UROS in transformed and wild tobacco plants is shown in Fig. 10B. ALAD and UROS activities did not differ among the three tobacco types, whereas PBGD activity was markedly lower in tobacco plants transformed with pFGC5941-*AbhemC*-RNAi than in the wild type and pFGC5941 vector-transformed plants. This suggests that inhibiting *hemC* expression results in decreased PBGD activity. In further analyses of the main Chl biosynthesis precursors, ALA and PBG concentrations did not differ among the three types of tobacco plant; however, Coprogen III, Proto IX, Mg-Proto IX, and Pchlide concentrations were markedly reduced in tobacco transformed with the pFGC5941-*AbhemC*-RNAi vector relative to the other two plant types (Fig. 10C*)*. These results show that decreased PBGD activity in turn inhibits the transition of PBG to Urogen III and then the lack of Urogen III results in reduced biosynthesis of the four mentioned precursors of Chl biosynthesis.

## Discussion

Leaf-color mutants provide excellent models for the study of Chl biosynthesis and degradation, chloroplast development, photosynthesis, and gene expression in plants (Dang et al., 1995; Larkin et al., 2003; Wang et al., 2012). As the most important photosynthetic pigment in plants, changes in Chl content can cause changes in leaf color (Chen et al., 2007). For example, in higher plants, inhibition of Chl biosynthesis reduces Chl content and leads to loss of leaf color (Koski & Smith, 1951; Reinbothe & Reinbothe, 1996; Rebeiz, 2013). In the present study, Chl a and Chl b concentrations in the Grs of chimeric leaves were 35.2-fold and 16.7-fold greater than the respective concentrations in the Whs; thus, Chl biosynthesis was suppressed significantly in the Whs. Chl biosynthesis in higher plants occurs via a series of continuous reactions in which ALA, PBG, Urogen III, Coprogen III, Proto IX, Mg-proto IX and Pchlide are the main synthetic precursors of these continuous reactions (Ilag, Kumar & Soll, 1994; Nagata et al., 2005; Shi, Liu & Jin, 2009). By determining the content of these synthetic precursors in leaves, it is possible to determine the step in the reaction in which Chl is inhibited (Liu, Chang & Du, 2015); this rate-limiting step in Chl biosynthesis differs among plant species. In the present study, the content of Urogen III, Coprogen III, Proto IX, Mg-Proto IX, and Pchlide in the Whs of chimeric *A. comosus* var. *bracteatus* leaves was strikingly lower than that in the Grs. Thus, reduced Chl biosynthesis in the Whs was likely caused by a shortage of Urogen III, which inhibited further synthesis of Chl. The conversion of PBG to Urogen III in the Chl synthesis pathway is therefore the first rate-limiting step in Chl biosynthesis in the Whs of chimeric leaves.

Previous studies have shown that the loss of leaf greenness is due to inhibited gene expression during Chl biosynthesis or in chloroplasts (Motohashi et al., 2003; Sugimoto et al., 2004; Chen, Bi & Li, 2005). Understanding the rate-limiting step in Chl biosynthesis is important for screening the key genes that play important roles in the albinism of leaf cells. Given that in the Whs of chimeric leaves this step was identified as the transition of PBG to Urogen III, it is likely that the enzyme PBGD, which catalyzes the transition of PBG to Urogen III, is important in Chl biosynthesis. To improve understanding of the molecular mechanism of albinism, it was therefore necessary to clone the PBGD gene and analyze its function. Our prokaryotic expression and in vitro enzyme activity analyses showed that we successfully integrated the cloned PBGD gene sequence and that the PBGD enzyme with catalytic activity was encoded. This enzyme is known to play an important role in Chl synthesis in plant cells (Witty et al., 1993). In the maize mutant *camouflage1*, PBGD defects can produce yellow-green leaves and necrosis (Huang et al., 2009). In a previous study, the *Arabidopsis rug1* mutant was found to be deficient in PBGD activity, with significantly increased accumulation of PBG when compared with wild-type plants (Quesada et al., 2013). In our research, the enzymatic activity of PBGD was reduced in the Whs of *A. comosus* var. *bracteatus* leaves but the level of PBG (the PBGD substrate) did not increase in these leaf areas. The disparity in these results may have arisen because *rug1* in *Arabidopsis* is a mutant,and the C →T change in the sequence of the gene encoding PBGD in *rug1* leads to the substitution of Ala →Val, which is a highly conserved residue in PBGD.So the disruption of the tetrapyrrole pathway at the step catalyzed by PBGD causes accumulation of PBG. Whereas we used a chimeric plant.

In transgenic plants, down-regulating an endogenous gene through an RNAi-mediated method is a powerful tool for analyzing gene function. Several studies have indicated that the efficacy of gene silencing is strongly related to self-complementary hairpin RNAs (Smith et al., 2000; Chen et al., 2003; Joseph, Ajisha & Jeevitha, 2012). In the current study, since *AbhemC* is highly homologous to *hemC* of tobacco, we cloned the common conserved region of the two genes into the pFGC5941 vector at the sense and antisense positions. Stable transgenic tobacco plants were generated with PPT screening after agrobacterium-mediated genetic transformation method using tobacco leaf disc. Firstly, RT-qPCR analysis showed that the transfer of *AbhemC*-RNAi significantly inhibited the expression of *hemC* in tobacco plants. Additionally, Chl and Urogen III content were much lower in the representative *AbhemC*-RNAi lines than in the controls. These findings were consistent with the pale-green phenotype of the transgenic tobacco plants. Moreover, PBGD activity decreased significantly in the transgenic lines relative to the control; thus, reduced *hemC* expression resulted in decreased PBGD activity, which disrupted PBG conversion to Urogen III and subsequently significantly reduced the content of other post-Urogen III precursor substances. Our comprehensive analysis therefore shows that *AbhemC* is an essential gene in Chl synthesis and albinism in *A. comosus* var. *bracteatus* leaves.

## Conclusions

In conclusion, we found that *AbhemC*, a gene that encodes PGBD, plays an important role in Chl biosynthesis and albinism in chimeric *A. comosus* var. *bracteatus* leaves. The conversion of PBG to Urogen III was the first rate-limiting step in Chl biosynthesis in the albino white parts of these leaves. The transformation of tobacco plants with pFGC5941-*AbhemC*-RNAi also suppressed *hemC* expression and reduced Chl content (as shown by pale green leaves), indicating that Chl synthesis was hindered in these plants. Furthermore, the suppression of *hemC* expression in these transgenic tobacco plants resulted in reduced PBGD enzyme activity, which in turn inhibited the transition of PBG to Urogen III, and likely led to decreased Chl content and the observed pale green-colored leaves.

##  Supplemental Information

10.7717/peerj.11118/supp-1 Supplemental Information 1 Details of the primer names and sequences used in this study

10.7717/peerj.11118/supp-2 Supplemental Information 2 Raw data for Figs. 3, 6, 7, 8 and 10.

## Additional Information and Declarations

### Data Availability

The following information was supplied regarding data availability: Raw measurements are available in the Supplemental Files.
