# Supplementary material for: AbhemC encoding porphobilinogen deaminase plays an important role in chlorophyll biosynthesis and function in albino Ananas comosus var. bracteatus leaves
Source: PeerJ. 2021 Mar 30;9:e11118. doi: 10.7717/peerj.11118 (PMC8018242; doi:10.7717/peerj.11118)
Supplement: Supplemental Information 1 [file peerj-09-11118-s001.docx]

| **Primer name** | **Primer sequence** |
| --- | --- |
| *AbhemC*  *AbhemC-1*  *18S rRNA*  *Actin*  *HemC-1*  *hemC-2* | F:5'-ACAATGGCGTGGCCCATCTCCATGA-3'  R:5'- TTAGTAAGCACGCAAACTACCAATC-3'  F: 5'- AATCGAAGAAGTCCTGGCCG-3'  R: 5'- CGAGTATTGGAGACGACGCA-3'  F: 5'-ATGGTGGTGACGGGTGAC-3'  R: 5'-CAGACACTAAAGCGCCCGGTA-3'  F:5'-TGATAACGGAACAGGAAT-3'  R:5'-CGACCAACAATACTAGGA-3'  F:5'-AATCGAAGAAGTCCTGGCCG-3'  R:5'-CGAGTATTGGAGACGACGCA-3'  F:5'-TCCCCACGTATGTCCCTGAT-3'  R:5'-CATAGCCAGCGATCGGAGTT-3' |
|  |  |

**Table S1 details of primers used in this study**
